# Accounting for uncertainty in health economic decision models by using model averaging

**DOI:** 10.1111/j.1467-985X.2008.00573.x

**Published:** 2009-04

**Authors:** Christopher H Jackson, Simon G Thompson, Linda D Sharples

**Affiliations:** Medical Research Council Biostatistics UnitCambridge, UK

**Keywords:** Akaike's information criterion, Bayesian information criterion, Health economics, Model averaging, Model uncertainty

## Abstract

Health economic decision models are subject to considerable uncertainty, much of which arises from choices between several plausible model structures, e.g. choices of covariates in a regression model. Such structural uncertainty is rarely accounted for formally in decision models but can be addressed by model averaging. We discuss the most common methods of averaging models and the principles underlying them. We apply them to a comparison of two surgical techniques for repairing abdominal aortic aneurysms. In model averaging, competing models are usually either weighted by using an asymptotically consistent model assessment criterion, such as the Bayesian information criterion, or a measure of predictive ability, such as Akaike's information criterion. We argue that the predictive approach is more suitable when modelling the complex underlying processes of interest in health economics, such as individual disease progression and response to treatment.

## 1. Uncertainty in health economic decision models

Health economic decision models are routinely used to guide the choice of the most appropriate treatment for patient groups on the basis of expected benefits and costs, commonly over a lifetime (National Institute for Health and Clinical Excellence, 2008). For chronic and recurring diseases, they are often implemented by using Markov models in which individuals move between clinical states of interest in discrete time periods, and each state is associated with a cost and benefit (Briggs *et al.*, 2006). The parameters of these models include probabilities governing transition between the states, the costs and benefits that are associated with each state and the effects of treatment and other covariates. Ideally, all available relevant evidence is used to inform these parameters, which may include randomized controlled trials and population mortality statistics. However, trials only provide information about relative effectiveness and costs of treatments in the short term, typically 5 years or less. To compare the treatments over patient lifetimes, extrapolations must be made, and the uncertainties that are inherent in the short-term results may be aggravated.

The expected costs and benefits for each treatment under the model, which are used to make the decision, are subject to uncertainty (Claxton *et al.*, 2002). In general, decision models are non-linear, so the expected model output does not equal the output evaluated at the expected values of the parameters of the model. Thus, to determine the expected costs and benefits accurately, it is necessary also to consider the uncertainty surrounding the inputs to the model, as discussed by Briggs *et al.* (2006), chapter 4. At the same time, considering the size and source of uncertainties can guide future research priorities (e.g. Tappenden *et al.* (2004)). Thus these uncertainties should be characterized, as discussed by Briggs (2000) and Bojke *et al.* (2006). Broadly, we distinguish between

- the choice of the most appropriate sources of data to inform the model (*judgement* uncertainty) and
- uncertainty about what inferences should be made from a particular set of data. Such *statistical* uncertainty can be further classified as

- parameter uncertainty—the choice of the specific values of parameters in a chosen model structure and
- model uncertainty—the choice of the appropriate model structure (Draper, 1995). In health economics, this might involve the set of clinical states to include, the choice of covariate effects to include on a particular transition probability, cost or benefit, or the choice of fixed or random-effects meta-analysis for synthesizing the results of trials (Bojke *et al.*, 2006).

Accounting for parameter uncertainty by probabilistic sensitivity analysis (Claxton *et al.*, 2002; Spiegelhalter and Best, 2003) is now well established. This involves placing probability distributions on the model parameters, often posterior distributions estimated by Bayesian methods. Monte Carlo simulation is then performed to estimate a distribution for the model outputs which accounts for the uncertainty in the inputs. In this paper, in contrast, we discuss methods of accounting for model uncertainty.

In health economics, although it is common to present a series of results under different structural assumptions, model uncertainty is rarely accounted for in a formal probabilistic manner. Russell (2005) recommended constructing a probability distribution over model structures, and Bojke *et al.* (2006) suggested that the model uncertainty be expressed through an extra parameter in the model during probabilistic sensitivity analysis. We describe how the required distribution over the choice of model structures can be obtained from the data. Essentially, this involves deriving weights from some measure of the adequacy of each model, judged against data. This leads to a model-averaged distribution for the model output as a weighted combination of the model-specific output distributions. Although measures of fit may be used to choose the best of several models which lead to different inferences, basing the decision purely on this best fitting model implies certainty that this model, and no others, is reasonable. In reality, there is rarely complete certainty. Weighting the outputs of the models according to the extent that the data support them should lead to better-informed decisions. Although the individual models themselves may be fitted from a Bayesian or classical perspective, we take a Bayesian view of the process of averaging the model outputs, considering the weights as *posterior model probabilities* for certain prior model probabilities.

In Section 2, we describe a decision model which compared two surgical methods of abdominal aortic aneurysm (AAA) repair, and we describe the main sources of statistical uncertainty in this model. In Section 3, we give a formal description of model averaging and review various model adequacy measures which can be used to weight the competing models. We discuss the underlying philosophies and principles behind each measure. Broadly, these are either geared towards assessing predictive performance or uncovering a ‘true’ data-generating mechanism. We argue that if the aim is to make predictions in situations where reality is complex, such as in health economic models for incidence of clinical events and response to treatment, then predictive model assessment is preferable. In this approach, more complex models are essentially given greater prior weight as the sample size increases. In Section 4, the model averaging methods are applied to the aneurysm surgery decision model. Finally, we suggest further extensions of the methods, and discuss how some other forms of model uncertainty in health economics may be addressed.

## 2. Application: surgery for abdominal aortic aneurysm repair

**Fig. 1:**
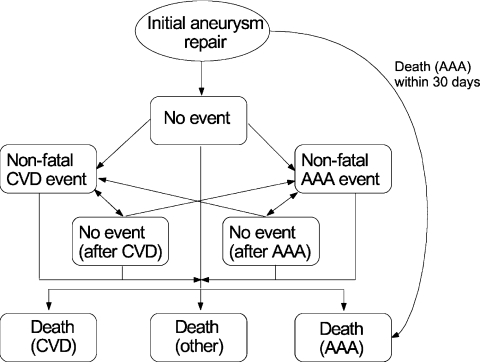
Representation of the Markov decision model for aneurysm repair: states and permitted transitions between them

The EVAR1 trial (EVAR Trial Participants, 2005) compared endo-vascular aneurysm repair (EVAR) with repair by open surgery in patients with large AAAs. Following the trial, which had an average follow-up of 3 years, a long-term model was developed (Epstein *et al.*, 2008) to assess the lifetime costs and benefits of EVAR compared with open repair for 74-year-old men. Following aneurysm repair, if patients survived at least 30 days after surgery, they were assumed to enter a long-term Markov model. This had eight states, which included hospital admissions for non-fatal AAA or other cardio-vascular disease (CVD) events, three states representing periods spent out of hospital and states representing death from three possible causes, illustrated in Fig. 1. In addition, a few patients receiving EVAR were converted to open repair during surgery. After conversion to open repair, surviving patients were assumed to enter a parallel long-term Markov model with the same states as in Fig. 1, but with some changed transition probabilities. The transition probabilities between the states were informed by data from the EVAR1 trial, population life tables and expert judgement. Further details of the model are given by Epstein *et al.* (2008). For example, the risk of death from CVD at any time for a patient receiving EVAR is the product of the mortality rate for CVD in the general population (from population data), the hazard ratio for CVD death among the trial population relative to the general population (from expert judgement) and the hazard ratio for EVAR compared with open repair (from the EVAR1 trial).

The standard framework was assumed for predicting expected costs and benefits, as follows. This is a discrete time, discrete state Markov model, with transition probability matrix *P*_*t*_, which evolves over *T*‘cycles’, or time units, *t* =1,…,*T*. The probability distribution of the state occupied by an individual at time *t* follows the recursive relationship *π*_*t*_=*π*_*t*−1_*P*_*t*_, where *π*_0_ is such that all individuals are in state 1 (no event) with probability 1 at time 0. There are costs *c*_*s*_,*s* =1,…,*S*, associated with one cycle spent in each of the *S* states and a fixed initial cost *c*_0_, and future costs are discounted at a rate of 100*δ*% per cycle. Then the total expected cost over *T* cycles for an individual undergoing a particular treatment is 
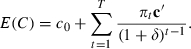


There is an analogous formula for the total expected benefit *E*(*B*). In this example, benefit was expressed in quality-adjusted life years (QALYs) (Torrance and Feeny, 1989), and the discount rate was 3.5% per year. The model is run twice, once assuming a surgical policy of EVAR and once with a policy of open repair. Estimation focuses on the incremental cost Δ_*C*_=*E*(*C*_1_)−*E*(*C*_0_) of EVAR compared with open repair, the incremental benefit Δ_*B*_=*E*(*B*_1_)−*E*(*B*_0_) and the incremental cost-effectiveness ratio (ICER) Δ_*C*_/Δ_*B*_, interpreted as the cost per QALY gained from using EVAR instead of open repair. A more effective new treatment (Δ_*B*_>0) is accepted if its ICER lies below a maximum value acceptable to the decision maker (Johannesson and Weinstein, 1993). The parameter uncertainty that is inherent in these quantities is accounted for by probabilistic sensitivity analysis, which provides simulated distributions of incremental costs and benefits. This leads to the probability PCE(*λ*) that EVAR is cost effective compared with open repair, defined as the probability of a positive incremental net benefit: 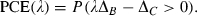
(1)

This depends on a ‘threshold’*λ*, the amount of money that a policy maker is willing to pay for 1 unit of benefit (such as 1 QALY).

The cost-effectiveness results for 74-year old men were presented by Epstein *et al.* (2008) for a plausible ‘base case’ followed by a series of eight alternative sets of reasonable model assumptions. The probability that EVAR was cost effective was substantially different from the base case under three alternative scenarios, defined by the following parameters.

- The first parameter is the mortality rate from CVD causes in the trial population compared with the general population. In the base case, this was assumed to be a hazard ratio of 2.00 (95% confidence interval 0.83–4.83). This was an expert judgement, loosely informed by the result of a previous study (Brady *et al.*, 2001). In the first alternative scenario, this hazard ratio was set to 1, an assumption of no effect.
- The second is the treatment effect on the CVD mortality rate. In the base case, this was taken to be a hazard ratio of 3.06 (95% confidence interval 1.12–8.36) for EVAR compared with open repair. This was calculated from a piecewise exponential survival model on the EVAR1 trial data and assumed only to operate in the second year after the surgery. In the second alternative scenario, this hazard ratio was set to 1.
- The third parameter is the treatment effect on the long-term AAA mortality rate. In the base case, this was a hazard ratio of 5.84 (0.70, 48.50) for EVAR, calculated from a Poisson regression of the EVAR1 trial data (six AAA deaths per 15132 person-months in the EVAR arm, *versus* 1 per 14720 in the open repair arm). In a third alternative scenario, this hazard ratio was set to 1.

Under four of the remaining five scenarios, the probability of cost-effectiveness was similar to the base case; therefore we do not consider these further in this paper. Cost-effectiveness results were also substantially different under a final alternative scenario in which mortality 30 days after open repair was 8%, obtained from routine hospital data, instead of the 5% that was observed in the EVAR1 trial data. This model choice is difficult to assess formally, since it involves judging which data are more representative of the population for which the policy will be made. Therefore we consider only scenarios (a)–(c) above in our formal quantification of model uncertainty in this paper.

The probability that EVAR is cost effective, PCE(*λ*), for *λ* =£20 000 per QALY, was estimated to be 0.011 under the base case. Under each of the three alternative scenarios, the incremental effectiveness of the EVAR treatment was higher; thus this probability was higher than under the base case: 0.020, 0.081 and 0.067. When all the hazard ratios were simultaneously set to 1, the probability that EVAR is cost effective was 0.52.

At the moment, the uncertainty arising from these three model choices can only be assessed by the reader informally ‘weighting’ the results by their opinion about the plausibility of the different scenarios. We aim to obtain a combined result which weights the individual results according to the extent to which the data formally support the models. The decision about the most cost-effective treatment will then take into account the uncertainty surrounding the model choice.

### 2.1 Characterizing covariate selection uncertainty

In statistical terms, these model choices are problems of *covariate selection* in regression. For example, choice (b) concerns whether to include treatment in a regression model for CVD mortality, or to assume no effect of treatment. Covariate selection could, alternatively, just be considered as parameter uncertainty. Then, predictions would be made from the largest model, using estimated posterior distributions of effects from a model containing all possible covariates. Some of these posterior distributions would be consistent with a covariate effect of zero. However, routine use of such large models with insufficient data to inform them would lead to poorer predictions (in a mean-squared error sense) and consequently less reliable decisions (Harrell *et al.*, 1996). Therefore, we consider covariate selection as uncertainty about model structure: the question is *which set of covariates do we include*, as well as *what are the effects of the included covariates and their uncertainties*?

Covariate selection problems are most often tackled in practice by searching for a combination of covariates with optimal combination of fit and parsimony, then basing inferences on that single model. This ignores the uncertainty that is involved in this selection, so the uncertainty about the eventual inference may be underestimated. As reviewed by Clyde and George (2004), methods have been proposed to account for the model selection uncertainty. In this paper, *model averaging* will be used to combine the results of models with different combinations of covariates, using weights derived from measures of model fit and parsimony. This technique is applicable to a wide range of model uncertainty problems as well as covariate selection.

## 3. Model averaging

Model averaging is a formal method of accounting for model uncertainty among predictions *y*_*k*_=*M*_*k*_(**x**) from a series of competing models *M*_*k*_,*k* =1,…,*K*, fitted to data **x**. In the Bayesian view of model averaging (Leamer, 1978; Draper, 1995; Kass and Raftery, 1995; Hoeting *et al.*, 1999) the interest is in the posterior predictive distribution of *y*. This is calculated as the average of the model-specific posterior predictive distributions over *posterior model probabilitiesp*(*M*_*k*_|**x**). 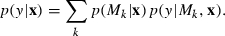
(2)

The probability that is assigned to model *k* is calculated as 
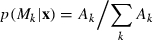
(3) where *A*_*k*_ is some adequacy measure for model *M*_*k*_, computed in terms of data **x**. If available, information on model adequacy external to the data may also contribute to *A*_*k*_ through *prior model probabilities*. Basing inferences purely on the model with the largest *A*_*k*_, or *model selection*, suggests complete certainty that this model is appropriate and the other models under consideration are inappropriate. In health economic contexts there is usually uncertainty about this choice, which model averaging takes into account.

### 3.1 Model adequacy measures

The likelihood is the fundamental measure of the fit of a model to data. However, the maximized likelihood will always increase with the number of parameters (such as covariate effects) in the model. As the number of parameters grows, the predictive variance of a fitted model increases. Therefore, a measure of adequacy is desired which makes a compromise between fit and complexity, or bias and variance.

Historically, two distinct adequacy measures have been used to compute probabilities for model averaging: *Akaike's information criterion* AIC and *marginal likelihood*. We now describe these measures in more detail. Although both of these trade off fit (measured by the likelihood) and complexity, they represent fundamentally different views of model assessment, as we discuss in Section 3.2.

#### 3.1.1 Akaike's information criterion

Suppose that a model *f*(*x*|*θ*) with parameters *θ* is fitted to data **x**, obtaining maximum likelihood estimates 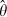. The expected Kullback–Leibler divergence from the truth of the predictive distribution of a replicate data set **y**, given this fitted model, is 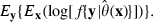


Akaike (1973) showed that the maximized log-likelihood 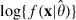 was an overestimate of this predictive discrepancy, because the parameters *θ* have been estimated. Using two second-order Taylor series approximations, this bias was shown to be asymptotically equal to *p*, the number of parameters in the model. The error of the approximation is *O*(1/√*n*), for sample size *n*, if the true process belongs to the same parametric family as *f* (Ripley, 1996). Hence, multiplying by the conventional −2, Akaike (1973) defined ‘an information criterion’ as 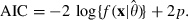
(4)

Thus, model selection based on minimum AIC seeks the model with the best predictive ability for a new data set generated by the same process, as measured by Kullback–Leibler divergence.

For model averaging, Buckland *et al.* (1997) and Burnham and Anderson (2002) set 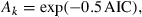
(5) thus transforming AIC back to the scale of probabilities. The resulting model probabilities, *A*_*k*_/Σ_*k*_*A*_*k*_, are often termed *Akaike weights*. Hjort and Claeskens (2003) rigorously assessed the properties of the resulting model-averaged estimators. All these researchers were working from a frequentist perspective and presented methods for calculating modified standard errors for the model-averaged outputs to account for model uncertainty.

#### 3.1.2 Marginal likelihood and Bayes factors

Bayesian model comparison and hypothesis testing are conventionally based on the *Bayes factor* or ratio of *marginal likelihoods* between models. See, for example, Kass and Raftery (1995) for a review of their theory, computation and interpretation. The marginal likelihood *f*(**x**|*M*) of a model *M* fitted to data **x** measures the ability of all model assumptions, both likelihood and prior, to predict the data **x**. It is defined by integrating the likelihood *f*(**x**|*θ*,*M*) with respect to the prior distribution *π*(*θ*|*M*) of parameters *θ*: 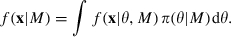
(6)

Commonly, model choice is based on maximizing the marginal likelihood, with the implicit assumption that the prior probabilities *p*(*M*) of all competing models are equal.

The marginal likelihood is difficult to compute in general. The integral is available only in closed form for some very simple cases such as linear regression (Raftery *et al.*, 1997), and other exponential family models with conjugate priors. Approximations are usually necessary. The most commonly used of these is a measure that is derived from Laplace integration, centred on the posterior mode or maximum likelihood estimate 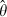 (Schwarz, 1978). Often called the Bayesian information criterion BIC, this measure is an asymptotic approximation to minus twice the logarithm of the marginal likelihood: 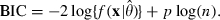
(7)*p* is the number of parameters, and *n* is the sample size. Note that this takes the same penalized log-likelihood form as AIC, but with a stricter penalty for complexity, which grows with the sample size.

As discussed by Kass and Raftery (1995), the ‘sample size’ is not always clearly defined. The term *n* in BIC arises from the Laplace integration via a further approximation: 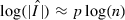, where 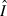 is the observed Fisher information matrix evaluated at the maximum likelihood estimate (Kass and Vaidyanathan, 1992). Informally, *n* is the number of units giving rise to a distinct piece of data, e.g. the number of observations in a normal distribution model, the sum of the denominators in a binomial logistic regression and the total number of counts in a Poisson log-linear model for a contingency table. In health economic models, Markov transition probabilities are often estimated by using Cox or parametric survival regressions. In these models, we take *n* to be the number of individuals, for consistency with logistic regression.

In Bayesian model averaging (Draper, 1995; Kass and Raftery, 1995) the weight for model *k* is usually defined as 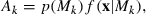
(8) where *p*(*M*_*k*_) is the prior probability over the model space that is assigned to model *k*, and *f*(**x**|*M*_*k*_) is the marginal likelihood of model *k*. Assuming equal prior model probabilities *p*(*M*_*k*_), the weights are therefore approximated as 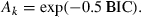
(9)

### 3.2. Principles behind AIC- and BIC-based model assessment

In Section 3.1, we described two classes of model adequacy measures that are used in model averaging, based on Kullback–Leibler predictive discrepancy and marginal likelihood, and their AIC- and BIC-approximations. These are based on fundamentally different principles. The choice of which of these measures to use depends on the purpose of the model assessment.

Firstly, observe that with BIC, if *n*≥8, there is a larger penalty for complexity (*ppt* log (*n*)), compared with AIC (2*p*). This penalty increases with the sample size *n*. This relationship of the marginal likelihood to the sample size ensures that model choice based on Bayes factors or BIC is *consistent* as the sample size increases. Suppose that, as *n* increases, the set of candidate models *M*_1_,…,*M*_*K*_ is fixed, and the priors on the parameters *π*(*θ*|*M*_*k*_) and the model space *p*(*M*_*k*_) are fixed. Then, as more data become available, there is some *k* such that the posterior model probability *p*(*M*_*k*_|**x**)→1 with probability 1 (Bernardo and Smith, 1994), i.e. selection based on marginal likelihoods converges to a single model choice as *n* increases. The advantage of this is that, if one of the candidate models is the true data-generating process, more data will always lead to uncovering that truth.

Conversely, model selection based on AIC will not consistently select the same model from a fixed set as the sample size increases. AIC aims to select the model with the best predictive ability for a future observation. As discussed by Burnham and Anderson (2002), as more data become available, better predictions will often result from larger models.

Bernardo and Smith (1994) discussed the notion of 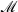-*closed* and 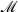-*open* model selection scenarios. In an 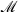-closed scenario, the set of candidate models is fixed in advance of data collection. In an 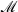-open scenario, the set of models under consideration is varied with the data: typically a wider range of models would be considered as the sample size increases. They argued that model comparison based on marginal likelihoods is only appropriate in an 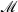-closed situation, where it is believed that one of the candidate models is the truth. This may be appropriate if there is a relatively low dimensional physical process generating the data, and the aim is to determine that process. In other circumstances, models are considered as convenient mechanisms to approximate highly complex processes. Then, model selection procedures based on predictive ability, such as cross-validation or AIC, are more appropriate. As discussed by Kadane and Lazar (2004), a compromise between the two approaches may sometimes be desirable, depending on the relative importance that is placed on predictive ability and model parsimony. In a Bayesian context, this compromise would involve varying the prior assumptions on the model space or the model parameters.

#### 3.2.1. Prior model probabilities implied by AIC

Another view on the principles underlying AIC is provided by interpreting model averaging by using AIC as a Bayesian procedure. Burnham and Anderson (2002) observed that AIC-based averaging (as in equation (5)) is equivalent to the conventional Bayesian model averaging procedure (as in equation (8)) using BIC to approximate *f*(**x**|*M*_*k*_), combined with specific implied prior model probabilities: 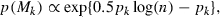
(10) i.e. the implied prior model probability depends on the sample size *n*, such that larger models (with a greater number of parameters *p*_*k*_) are more likely to be considered when there are more data. They argued that this is usually preferable, since in real applications the truth is usually complex. Although these implied priors might seem strongly to favour complexity (e.g. *p*(*M*_1_)=0.08 and *p*(*M*_2_)=0.92 for *n* =1000 and *p*_1_=1 and *p*_2_=2), their influence is moderated when combined with the BIC-based ‘model likelihood’, which has a heavy complexity penalty. If there is additional information, external to the data, about the preference between models, this could be used to weight the *p*(*M*_*k*_) of equation (10). Then the model weights would be based on both prior information and predictive ability judged from the data.

Burnham and Anderson (2002) also compared the predictive ability of AIC- and BIC-based model selection by simulation. Model selection using BIC (and implied equal prior model probabilities) was shown to give a lower mean-square predictive error than AIC when there was a low dimensional ‘true model’ with only a few large effects. AIC performed better when the truth was a more complex model with a few large effects and many small effects. Model-averaged predictions, using either method, were consistently better than predictions that were conditional on a selected ‘best’ model.

### 3.3. Implementation and consequences of model averaging

Implementation of Bayesian model averaging and its consequences for inference were discussed in detail by Hoeting *et al.* (1999). One important issue is how to choose the set of *candidate* models to be averaged over. Draper (1995) recommended that candidate models should be chosen to ‘stake out the corners in the model space’, i.e. a set of reasonably well-supported models with different predictive consequences should be considered: there is no point in averaging over a set of several models which lead to very similar inferences, even though they all have very similar fits to the data. When averaging over models which give different predictions, the model-averaged inferences will generally have greater uncertainty than the model-specific inferences. Conversely, as remarked by Tukey (in the discussion of Draper (1995)), when averaging over two models with identical point estimates, one with tight confidence limits and the other with wider limits, the variance of the averaged prediction will be between the two model-specific variances.

In general, computing posterior model probabilities for model averaging is easy by using AIC or BIC approximations: only a maximized likelihood for each competing model is required, which is outputted by standard software. Furthermore, for a model choice between two models with and without a particular covariate, as in the aneurysm surgery example, there is an even simpler way of computing posterior model probabilities. As we now show, a *p*-value for the covariate effect is all that is required.

#### 3.3.1. Posterior model probabilities for a single-covariate selection problem

For either model adequacy criterion, *A*_*k*_= exp (−*a*_*k*_/2), where *a*_*k*_ is either AIC or BIC, the posterior model probability in equation (3) can be rewritten as 
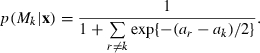


Therefore the *difference* in *a*_*k*_ between model *k* and each other candidate model *r* is sufficient to be able to calculate the posterior probability of model *k*. Recalling the definition of AIC (equation (4)), for a choice between two models *M*_1_ and *M*_2_, the *likelihood ratio* between the models is sufficient information to be able to calculate the difference in AIC, ΔAIC, and therefore the posterior model probabilities. BIC also requires the sample size *n*.

Given a published covariate effect and confidence interval or standard error (as in Section 2), the resulting two-sided Wald *p*-value, **p**, can be computed. Hence this likelihood ratio can be estimated by assuming that this *p*-value is asymptotically equal to the 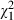
*p*-value of minus twice the log-likelihood ratio of *M*_2_ and *M*_1_. For example, in our model choice (b) in Section 2, we label the model with no treatment effect on CVD mortality as *M*_1_, and the model including this effect as *M*_2_. The posterior probability of the model with the treatment effect, under AIC-based model averaging, is then 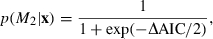 where 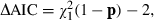 the AIC without the covariate minus the AIC with the covariate, and 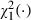 is the inverse cumulative distribution function of the 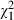-distribution. Similarly, under BIC-based averaging, 
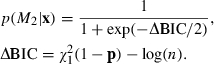


The advantage of calculating the posterior model probability in this way is that individual level data are not required: merely a published effect and standard error or confidence interval. Individual level data would be needed if we wished to account for the uncertainty surrounding inclusion of two or more covariates in a single regression. Then, the published estimate and confidence interval for each covariate would not be sufficient to calculate the likelihood ratios between every pair of regression models with every (plausible) combination of covariates.

**Fig. 2:**
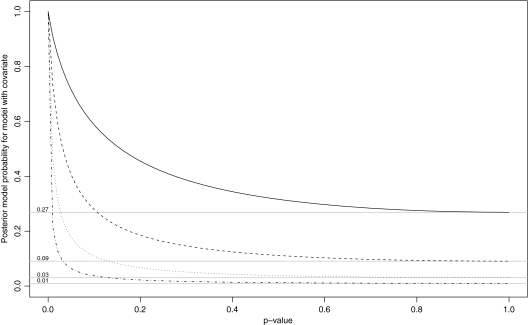
Posterior model probabilities for a single-covariate model choice, in terms of covariate *p*-values, for AIC- and BIC-based model averaging (the minimum probabilities, achieved when *p* =1, are indicated as faint horizontal lines): 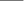, AIC; 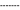, BIC (*n* =100); 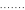, BIC (*n* =1000); · - · - · -, BIC (*n* =10 000)

Fig. 2 illustrates how the posterior model probability that is assigned to the model with the covariate decreases as the likelihood ratio *p*-value for that covariate increases, under AIC-based model averaging and BIC-based model averaging with sample sizes of 100, 1000 and 10000. Whatever the *p*-value, the likelihood of the more complex model cannot be less than the likelihood of the simpler model; therefore ΔAIC≥−2 and *p*(*M*_2_|**x**)≥(1+e)^−1^=0.27. AIC-based model averaging always gives reasonable weight to the more complex model, considering that there is always some chance that the study was underpowered to detect the effect of the covariate. Conversely, as the sample size increases, BIC gives increasingly less weight to the covariate, even for those with conventionally ‘significant’ low *p*-values.

## 4. Application to surgery for aneurysm repair

We now return to the aneurysm surgery cost-effectiveness study that was introduced in Section 2. We apply the Bayesian model averaging techniques that were discussed in Section 3 to calculate posterior predictive distributions of incremental lifetime cost and effectiveness, accounting for model uncertainty. In this study, there are three covariate selection choices, each considering two competing models, leading to 2×2×2=8 combinations of models. We calculate four sets of model-averaged estimates, over different sets of candidate models as follows:

- over the base case and a single alternative scenario, for each of the three alternative scenarios that were described in Section 2;
- over all eight combinations.

Since mortality among aneurysm patients is a highly complex process, we believe that a model adequacy measure that is based on predictive ability is more appropriate than one geared towards determining the true data-generating mechanism. Therefore we focus on using AIC, as in equation (5), to compute posterior model probabilities. We compare these with model-averaged results that are obtained by using equation (8); specifically, we take the BIC approximation (7) to *p*(*M*_*k*_|**x**) combined with prior model probabilities *p*(*M*_*k*_) equal for all candidate models (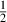 when averaging two models, and 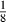 when averaging eight models).

For covariate choices (b) and (c) in Section 2, the treatment effects on CVD and AAA mortality rates respectively, the sample size used in the calculation of BIC was *n* =1016, the number of individuals in the trial data on which the hazard ratios had been estimated. For covariate choice (a), the relative hazard of CVD mortality between AAA patients and the general population, we take the sample size from the study by Brady *et al.* (2001), as *n* =1139, which was the principal source of data used to inform this parameter.

### 4.1. Results: posterior model probabilities

Table 1 presents *p*-values, AIC and BIC differences and resulting posterior model probabilities for each of the three covariate choice problems that were described in Section 2. The positive differences in AIC indicate that the base case model with the covariate is preferred in all three choices by AIC. The negative differences in BIC indicate that BIC-based model selection, which incurs a stronger penalty for model complexity, prefers the alternative simpler model without the covariate in all three choices. The simpler model is even preferred under the second choice: whether there is a treatment effect on the CVD death rate, for which the 95% lower confidence limit for the hazard ratio exceeded 1.

**Table 1:** Summary of the three model choice problems†

*Parameter*	*Hazard ratio(95%interval)*	*2-sided p-value*	*Sample size n*	*Difference in the following criteria:*		*Posterior probability of model with covariate*	
				*AIC*	*BIC*	*AIC*	*BIC*
(a) Difference between trial and population in CVD death hazard	2.00 (0.83, 4.83)	0.123	1139	0.373	−4.621	0.546	0.090
							
(b) Treatment effect on CVD deaths	3.06 (1.12, 8.36)	0.029	1016	2.785	−2.138	0.801	0.256
(c) Treatment effect on AAA deaths	5.84 (0.70, 48.50)	0.102	1016	1.826	−3.098	0.714	0.175

† The base case in each problem includes the covariate; the alternative case excludes the covariate.

Thus, the posterior probabilities for the models with the covariate are all greater than 0.5 under AIC assessment, and less than 0.5 under BIC assessment. The posterior probability of a non-zero covariate effect is highest for the second choice.

### 4.2. Results: model-averaged cost-effectiveness analysis

These posterior model probabilities are now used to perform model-averaged cost-effectiveness analyses. Bayesian model averaging and probabilistic sensitivity analysis are combined as follows. To produce a sample of size *N* from the posterior predictive distribution of expected incremental cost and effectiveness, averaged over models *M*_1_ and *M*_2_, *N* *p*(*M*_1_|**x**) Monte Carlo replicates from model *M*_1_ were merged with *Np*(*M*_2_|**x**) replicates from model *M*_2_. In the probabilistic sensitivity analysis, to be consistent with the published analysis (Epstein *et al.*, 2008) under the base case model, log-normal probability distributions were assigned to all hazard ratios (parameter (a) and (b) in Section 2) and gamma distributions assigned to the event rates (parameter (c)). Means and variances correspond exactly to the estimates and confidence intervals that were presented in Section 2. Under the alternative assumptions (a)–(c), the corresponding hazard ratio is assumed to be 1 with zero variance. *N* =5000 total Monte Carlo replicates were used (note that our results do not exactly match those presented by Epstein *et al.* (2008), who used 1000 replicates).

**Fig. 3:**
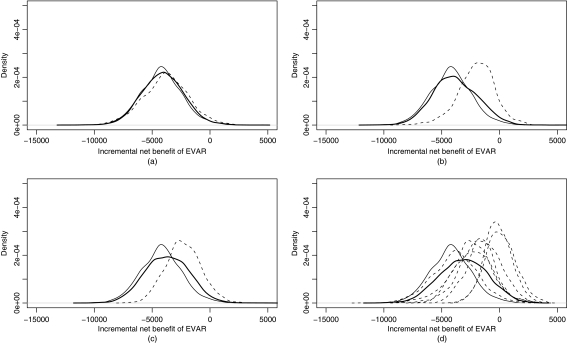
Kernel density estimates of the posterior predictive distributions of the expected incremental net benefit of EVAR in pounds, assuming a will- ingness-to-pay threshold of *λ* =£20 000, for four sets of AIC-based model-averaged analyses (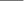, with covariate; 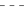, without (some) covariate(s); 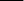, model average): (a) base case and assumption (a); (b) base case and assumption (b); (c) base case and assumption (c); (d) all eight averaged

**Fig. 4:**
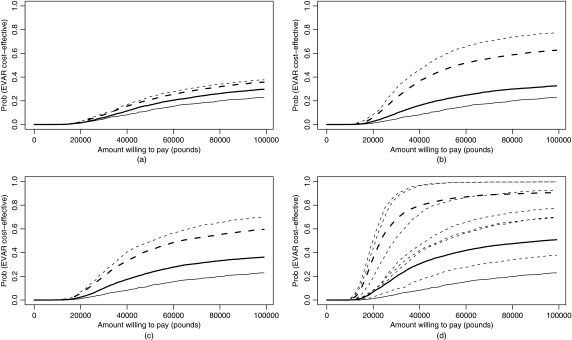
Cost-effectiveness acceptability curves for the base case (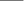), alternative assumptions (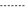) and AIC (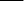) and BIC (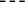) model averages: (a) base case and assumption (a) averaged; (b) base case and assumption (b) averaged; (c) base case and assumption (c) averaged; (d) all eight averaged

Posterior means and 95% credible intervals for expected incremental costs and expected QALYs gained are presented in Table 2. Kernel density estimates of the posterior predictive distribution of incremental net benefit (for a willingness-to-pay threshold of *λ* =£20 000 per QALY) are illustrated in Fig. 3, for AIC-based model averaging. Cost-effectiveness acceptability curves, which plot the probability of positive incremental net benefit (equation (1)) against *λ*, are presented in Fig. 4 for AIC- and BIC-based averaging.

**Table 2:** Cost-effectiveness analyses for single models and averaged combinations of models: posterior means and 95% credible intervals for expected incremental cost, incremental QALYs of EVAR compared with open repair, incremental cost-effectiveness ratio and probability of cost-effectiveness PCE(*λ*) for thresholds of *λ* =£20 000 and £40 000 per QALY†

	*Incremental cost* (Δ_*C*_)(£)	*Incremental QALY* (Δ_*B*_)	*ICER* (Δ_*C*_/Δ_*B*_)	*PCE* (£20000 *threshold)*	*PCE* (£40000*threshold*)
*Single models*					
Base case 0	3790	−0.023	Negative	0.011	0.079
	(2410, 5230)	(−0.19, 0.15)			
Assumption (a)	4130	0.012	353000	0.020	0.15
	(2780, 5580)	(−0.18, 0.20)			
Assumption (b)	3710	0.083	44800	0.081	0.46
	(2320, 5160)	(−0.065, 0.22)			
Assumption (c)	3870	0.075	51400	0.067	0.38
	(2500, 5300)	(−0.081, 0.23)			
Assumptions (a) and (b)	4070	0.079	51400	0.076	0.42
	(2680, 5500)	(−0.11, 0.24)			
Assumptions (a) and (c)	4210	0.14	29200	0.18	0.71
	(2850, 5600)	(−0.0018, 0.28)			
Assumptions (b) and (c)	3790	0.18	20900	0.43	0.97
	(2400, 5200)	(0.085, 0.29)			
Assumptions (a)–(c)	4150	0.21	19800	0.52	0.97
	(2770, 5560)	(0.099, 0.33)			
*AIC-based veraging*					
Base case and assumption (a) averaged	3940	−0.0072	Negative	0.013	0.12
	(2560, 5380)	(−0.18, 0.18)			
Base case and assumption (b) averaged	3770	−0.0028	Negative	0.026	0.16
	(2400, 5170)	(−0.18, 0.18)			
Base case and assumption (c) averaged	3800	0.0051	749000	0.024	0.17
	(2450, 5210)	(−0.18, 0.19)			
All eight averaged	3950	0.043	92800	0.069	0.30
	(2560, 5420)	(−0.16, 0.24)			
*BIC-based veraging*					
Base case and assumption (a) averaged	4110	0.008	512000	0.021	0.16
	(2720, 5480)	(−0.18, 0.20)			
Base case and assumption (b) averaged	3720	0.056	66600	0.062	0.37
	(2350, 5100)	(−0.14, 0.21)			
Base case and assumption (c) averaged	3850	0.057	67500	0.056	0.33
	(2510, 5220)	(−0.12, 0.22)			
All eight averaged	4110	0.17	24900	0.35	0.80
	(2750, 5570)	(−0.052, 0.31)			

† ‘Negative’ ICER indicates that EVAR was more costly and less effective on average.

Firstly, a set of model-specific (not model-averaged) results are presented for reference. These are presented for the base case which includes all covariates, each of the three alternative scenarios which exclude one covariate and combinations of the alternative scenarios which exclude more than one covariate. In the tables and figures, assumptions (a), (b) and (c) refer to the three alternatives described in Section 2: baseline hazard of CVD death the same as the general population, no treatment effect on CVD death hazard and no treatment effect on AAA death hazard respectively.

Secondly, three sets of model-averaged results are calculated: one for each model choice, averaging over the base case and each of the three alternative scenarios in turn. This shows the effect of accounting for one source of model uncertainty at a time. Finally, an overall result, averaged over the eight combinations of assumptions implied by the three model choices, is presented, which accounts for all three sources of model uncertainty simultaneously. The three choices are assumed to be independent, so that the posterior model probability for a combination of models is the product of three independent model probabilities.

The alternative assumptions, in which one of three covariate effects in turn is assumed to be null, all produce a higher probability of cost-effectiveness than the base case model containing all the covariates. In addition, when more than one of the covariate effects is assumed to be null, the probability that EVAR is cost effective is even higher. The highest probability of cost-effectiveness at a threshold of £20 000 per QALY is about 50% when it is assumed that there is no difference between the trial and general population in CVD mortality, and no treatment effect on either the CVD or AAA mortality rates.

By model averaging, we take into account our uncertainty about whether to include these covariate effects in the model. AIC-based model averaging favours the base case, i.e. the model with non-zero covariate effect. Fig. 4 illustrates that, when the AIC is used to calculate posterior model probabilities, the model-averaged cost-effectiveness probability is closer to the base case assumption than the null alternative. In contrast, BIC prefers the simpler model: the BIC model averaged cost-effectiveness acceptability curves are generally closer to those which assume no effect. Thus the probability of cost-effectiveness at £20 000 per QALY, averaged over all models by using AIC, is 0.069, compared with 0.35 for the same probability averaged by using BIC, and 0.011 in the original base case.

The uncertainty surrounding the model-averaged predictions is higher in situations where the model-specific results being averaged over are different. For example, the base case and assumption (b) give very different estimates of incremental QALY (−0.023 and 0.083 respectively). The credible interval surrounding the averaged estimate of −0.0028, and thus the incremental net benefit, is wider than either of the model-specific credible intervals, though not substantially (Fig. 3(b)). Similar behaviour is seen when averaging the base case and assumption (c), and when averaging all eight combinations of models. In contrast, the estimates of incremental cost from the base case and alternative assumptions, and the uncertainty surrounding them, are not very different; thus the resulting model-averaged estimates of cost and their credible intervals are also similar (Table 2).

We briefly assessed the sensitivity of the model-averaged result to the assumption that the model choices are independent. There is no reason to believe that the hazard ratio between the trial and general population in the CVD mortality rate may be correlated with the hazard ratio of treatment on either CVD or AAA mortality. However, the second and third model choices may not be independent, since treatment may affect AAA and CVD mortality in a similar way. If these were positively correlated, then the results under the scenarios in which alternatives (b) and (c) both hold would be underweighted, and the results under scenarios where only one holds would be overweighted. Individual level data would be needed to estimate this correlation. In the absence of such data, we performed a sensitivity analysis in which the probabilities of alternatives (b) and (c) were perfectly correlated. Under this assumption, the model-averaged probability of cost-effectiveness at a threshold of £20000 per QALY increased from 0.07 to 0.11.

### 4.3. Substantive conclusions

Estimated probabilities that EVAR is cost effective compared with open repair, for a threshold of £20000 per QALY, ranged from 0.01 under the most probable base case, to the alternative of 0.081 where one covariate effect was omitted, to 0.52 under a scenario where all covariate effects were omitted. Although it is fairly clear, from considering the relative plausibility of these scenarios, that EVAR is not conventionally cost effective, there seems to be considerable uncertainty surrounding the exact probability of cost-effectiveness. Using Bayesian model averaging based on AIC, we obtained an estimate of 0.069 for this probability, which forms a statistically principled compromise between the alternative assumptions.

We note that Epstein *et al.* (2008) presented another plausible alternative scenario, in which the 30-day mortality after open repair was 8% (from routine hospital data) instead of 5% (from the EVAR1 trial data). Under this scenario, the cost-effectiveness of EVAR at £20000 per QALY was 0.147 (ICER £42000 per QALY). To include this scenario in a model-averaged analysis, an expert assessment of the plausibility of each alternative mortality rate would be required, i.e. an assessment of how representative each alternative data source is of the population for which the policy will be implemented.

## 5 Discussion

### 5.1. Conclusions

Bayesian model averaging can be used to account for uncertainty about health economic model structure. This allows a set of results that are obtained under alternative scenarios to be explicitly weighted according to their fit to data, instead of the decision maker implicitly weighting them according to an informal judgement. Accounting for model uncertainty enables better-informed decisions about the most cost-effective treatment choice. Model uncertainty is particularly important in decision problems which involve long-term extrapolation, since any inaccuracies in models that are fitted to short-term data will be magnified when used for long-term prediction. If there are several scenarios which are supported by the data but give different predictions, then averaging over them can give improved estimates with more honest uncertainty intervals. Although we illustrated its use for covariate choice, we envisage that model averaging may also be useful for other common model uncertainties in health economics, such as the choice between fixed and random-effects meta-analysis, or the shape of the relationship of mortality to age.

We emphasize that the methods that are discussed in this paper can only account for *statistical* uncertainties, i.e. uncertainties which can be assessed against data. Uncertainties about judgements are equally important in health economics. These might include the choice of the most appropriate studies of the treatment to inform the model, assumptions about how to generalize the results of a study of one population to a different population or discount rates. As these models involve extrapolating many years into the future, perhaps the most important judgements are assumptions about potential changes in parameters, such as treatment effects and costs. Data to inform such assumptions are not generally available. To account for these types of uncertainty most accurately, the beliefs of experts should be elicited rigorously, as discussed, for example, by O'Hagan *et al.* (2006). Model averaging, as in equation (2), may still be applied in these situations, but with the model probabilities *p*(*M*_*k*_|**x**) determined purely from prior beliefs instead of the fit of model *k* to data.

Model averaging is intended to supplement, rather than to replace, deterministic sensitivity analyses in which results are presented under different scenarios. The scenario-specific results are still important to illustrate the influence on the decision and research priorities if beliefs about certain parameters were to change in the light of new evidence. Expected value of partial perfect information (Welton *et al.*, 2008) is a formal method for calculating the decision uncertainty that is associated with each parameter, thus prioritizing what new evidence should be collected. This can be implemented within the probabilistic framework that we use.

Using AIC or BIC approximations, model averaging may be applied easily as part of routine probabilistic sensitivity analysis. It requires merely a maximized likelihood for each competing model, which is presented by most statistical software, and no further computer-intensive calculations, such as Markov chain Monte Carlo (MCMC) sampling. Indeed, in the very simplest case of averaging over two models with and without a covariate, only a *p*-value for that covariate is required to estimate posterior model probabilities. However, the results are dependent on the assumptions underlying the model assessment measure that is used to weight the competing models. When sample sizes are reasonably large, as in our example where *n* was about 1000, AIC-based model averaging gives substantially more weight than BIC-based averaging to more complex models. We believe that when the main purpose of modelling is to make predictions based on a complex reality, as in our health economic context, then a measure that is based on predictive ability, such as AIC, is more appropriate. Model assessment methods that are based on marginal likelihood, such as BIC, are more suitable where it is believed that there is a relatively simple true model underlying the data, and the purpose of modelling is to determine that mechanism.

### 5.2. Further developments in model uncertainty

Our application emphasized simple, routinely applicable methods for accounting for model uncertainty in health economic decision problems. There are many potential variations of these basic techniques which may be more appropriate in other situations. For example, in smaller samples, the asymptotic approximations involved in AIC and BIC may not be appropriate.

#### 5.2.1. Extensions of AIC and BIC model adequacy principles

Many extensions of the principles of AIC have been proposed. Some have sought to improve the approximation of AIC to the underlying Kullback–Leibler divergence, e.g. TIC (Takeuchi, 1976), GIC (Konishi and Kitagawa, 1996), the small-sample bias-corrected AIC_c_ (Hurvich and Tsai, 1989,^1995^) and the bootstrap-based EIC (Ishiguro *et al.*, 1997). KIC (Cavanaugh, 1999) aimed to correct AIC for the asymmetry of the Kullback–Leibler distance between two distributions. Spiegelhalter *et al.* (2002) derived the *deviance information criterion* DIC as a generalization of AIC to hierarchical models where the number of parameters *p* is not well defined. Since Bayesian hierarchical models are becoming more common in health policy evaluation, we would welcome investigation into whether DIC can be used as a basis for model averaging. NIC (Murata *et al.*, 1994) was defined with a similar aim of assessing the complexities of neural network models. Claeskens and Hjort (2003) defined a *focused information criterion* FIC, which was geared towards optimal estimation of the particular parameter, or function of parameters, of most interest. The risk inflation criterion RIC (Foster and George, 1994) for linear model covariate selection aims to minimize maximum predictive risk due to selection.

When applying marginal likelihood for small samples, it would be preferable to use a more accurate approximation than BIC (Kass and Raftery, 1995; Han and Carlin, 2001). In our example, the model parameters were estimated by maximum likelihood but, if fully Bayesian inference is employed, one drawback of marginal likelihood is its sensitivity to the prior distribution *π*(*θ*|*M*) for the parameters. This poses a problem if there is genuinely weak prior information. Indeed, under improper priors, the marginal likelihood is undefined. This has motivated several variants of Bayes factors. The BIC-approximation either implicitly disregards priors for the parameters, in which case it provides an *O*(1) approximation to the marginal likelihood, or assumes a ‘unit information’ reference prior (Kass and Wasserman, 1995) under which it has an *O*(*n*^−1/2^) error. A unit information prior has precision that is equivalent to the information that is available in one observation. Geisser and Eddy (1979) derived a *pseudo*-Bayes factor by replacing the likelihood by a cross-validatory predictive density. The *posterior* Bayes factor (Aitkin, 1991) is derived by replacing the prior by the posterior in the definition of the marginal likelihood. The *fractional* Bayes factor (O'Hagan, 1995) and *intrinsic* Bayes factor (Berger and Pericchi, 1996) are based on the principle of reserving part of the data to convert an improper prior into a proper posterior, and using this posterior as a prior to compute a conventional Bayes factor for the remainder of the data.

Gelfand and Dey (1994) derived asymptotic approximations to some of these alternative Bayes factors which are analogous to the BIC. Whereas BIC implies that, in the Bayes factor, the minus twice the log-likelihood is penalized by approximately *p* log (*n*), the pseudo-, posterior and intrinsic Bayes factors imply penalties of *p*,*p* log (2) and *p* log (*n*) respectively. We would expect variants of marginal likelihood with a predictive justification, e.g. the pseudo-Bayes factor, whose implicit complexity penalty does not depend on sample size, to produce model assessments similar to AIC, whose penalty is 2*p*. This may be a reasonable alternative to AIC for fully Bayesian model averaging. Similarly, Stone (1977) showed that AIC model choice was asymptotically equivalent to frequentist cross-validation. In general, computationally intensive methods of model selection, involving techniques such as cross-validation or bootstrap resampling, can improve on simple criteria, as discussed by Hastie *et al.* (2001) in the context of machine learning. For example, in the ‘stacking’ method of model averaging (Wolpert, 1992), the weights comprising the model-averaged prediction are chosen to minimize cross-validatory squared error.

#### 5.2.2. Averaging over large numbers of models

Often it is desired to consider large numbers of competing models. For example, when selecting between 10 potential covariates, then there are 2^10^, over a 1000, candidate models. The *Occam's window* principle and algorithm (Madigan and Raftery, 1994) aims to choose a manageable set of models which are both parsimonious and supported by the data. Firstly, models with less than a certain posterior probability are not considered and, secondly, more complex models which receive substantially less support than nested simpler models are not considered. An arbitrary threshold must be chosen when applying both principles. Madigan and York (1995) described MCMC methods to approximate expression (2), when this involves a very large number of candidate models *M*_*k*_.

#### 5.2.3. Markov chain Monte Carlo sampling over the model space

An alternative to marginal likelihood for computing posterior model probabilities in a fully Bayesian setting is reversible jump MCMC sampling (Green, 1995). Its advantage is that within-model parameter estimates, posterior model probabilities and model-averaged posterior predictive distributions can all be calculated simultaneously in a single MCMC run, even when there are different numbers of parameters in each model. The MCMC sampler moves simultaneously in the model space and parameter space, and model comparison is based on Bayes factor principles. Han and Carlin (2001) reviewed various MCMC methods for computing posterior model probabilities, including marginal likelihood and the reversible jump.

#### 5.2.4. Continuous model uncertainty

An attractive alternative to averaging over a discrete set of candidate models, which was recommended by Draper (1995) and Gelman *et al.* (2003), is to consider model uncertainty as continuous. This involves constructing, if possible, a very general model, which includes all the models under consideration as special cases. The models under consideration are defined by values of a continuously varying parameter in the general model. If there is a choice between different parametric families (e.g. a choice between a log-normal or gamma distribution for skewed cost data) then Bayesian non-parametric methods (e.g. Ohlssen *et al.* (2007)) may be necessary to build an expanded model.

For example, in the context of covariate selection, one single model containing every covariate may be considered. Since the true effects of some covariates may be very small or zero, a prior distribution is placed on the covariate effects. An informative prior could be used to constrain parameters about which the study data contain little information, and to ensure that the posterior distributions are consistent with expert belief. Alternatively, a hierarchical prior could be based on the data alone, to improve predictive precision by shrinking the possibly unnecessary coefficients towards 0. Greenland (1993) and Witte and Greenland (1996) took this approach in the context of multiple exposures in epidemiology. For example, a prior distribution *π*(*β*|*τ*^2^)=*N*(0,*τ*^2^) could be placed on the covariate effect *β*, and *τ*^2^ could be estimated by using the empirical Bayes procedure of maximizing the likelihood integrated over the covariate effect: ∫*f*(**x**|*β*,…) *π*(*β*|*τ*^2^) d*β*. *Ridge regression* (e.g. Draper and Smith (1998)) is a similar technique for shrinking regression coefficients.

Model averaging methods essentially consider the prior for the covariate effect to be a mixture of a point mass on zero and a continuous distribution excluding zero. The above method is a smoother alternative. Different methods of choosing such a smooth prior would be analogous to the different assumptions that are involved in choosing a model adequacy measure to use for model averaging. For example, George and Foster (2000) showed that certain priors for normal linear regression coefficients led to selection criteria that are equivalent to AIC and BIC. However, it may be reasonable to give special prior privilege to a covariate effect of zero: some would consider it a major difference in interpretation from stating that a treatment or risk factor has a very small effect on a disease to stating that it has zero effect.
